# HER4 expression status correlates with improved outcome in both neoadjuvant and adjuvant Trastuzumab treated invasive breast carcinoma

**DOI:** 10.18632/oncotarget.1232

**Published:** 2013-08-26

**Authors:** Bryce P Portier, Eugen C Minca, Zhen Wang, Christopher Lanigan, Aaron M Gruver, Erinn Downs-Kelly, G Thomas Budd, Raymond R Tubbs

**Affiliations:** ^1^ Department of Pathology and Genomic Medicine, Houston Methodist, Houston, TX; ^2^ Pathology and Laboratory Medicine Institute, Cleveland Clinic, Cleveland, OH; ^3^ Department of Solid Tumor Oncology, Taussig Cancer Institute, Cleveland Clinic, Cleveland, OH

**Keywords:** Breast carcinoma, HER2, HER4, Immunohistochemistry, RT-qPCR, Trastuzumab, H-Score

## Abstract

Prognostic and predictive markers utilized in invasive breast carcinoma are limited and include ER, PR, Ki67, and *ERBB2* (HER2). In the case of HER2, over-expression or amplification serves as eligibility for anti-HER2 based therapy, including trastuzumab (Herceptin®, Genentech). While clinical trials have shown trastuzumab improves overall survival and time to progression, an individual's response to anti-HER2 based therapy is highly variable. This suggests that, in a “uniform” HER2 positive population, additional markers could help in predicting patient outcome to therapy. Here we utilized a recently validated high-specificity HER4 antibody (E200) and generated a standard clinical HER4 scoring algorithm (HER4 H-Score) utilizing two breast carcinoma cohorts: 1) patients receiving neoadjuvant trastuzumab (n=47) and 2) patients receiving trastuzumab for metastatic disease (n=33). Our HER4 H-Score showed significant correlation with high sensitivity RT-qPCR performed on matched patients (p=<0.0001). In addition, patients with HER2/HER4 co-over-expression status showed a significant delay in development of metastasis after neo-adjuvant trastuzumab therapy (p= 0.04) and showed a significant improvement in progression free survival after adjuvant trastuzumab therapy (p=0.03). These findings suggest HER4 IHC, used in conjunction with a standard HER2 testing algorithm, could aid in predicting clinical outcome and help identify patients likely to show improved response to trastuzumab therapy.

## INTRODUCTION

*ERBB2* (HER2) is a well-characterized membrane receptor in the EGFR family and a therapeutic target in invasive breast carcinoma. Targeted anti-HER2 therapy with trastuzumab in patients with HER2 over-expression or amplification improves overall survival and recurrence free survival [1]. While HER2 over-expression/amplification is a prerequisite for patient eligibility to receive anti-HER2 based therapy, an individual's response to such treatment is highly variable. Some HER2 positive patients have essentially no response while others may achieve a complete response and/or remission [2-8]. This differential response cannot be solely attributed to discrepancies in expression and amplification status as determined by standard laboratory HER2 testing, including immunohistochemistry (IHC) and fluorescence in situ hybridization (FISH) methodologies [9]. In a “uniform” population of HER2 positive cases, it is reasonable to hypothesize that refined outcome prediction can be achieved by assessing alternative biomarkers.

Candidate markers for refining predicted outcome post trastuzumab therapy include the remaining EGFR family members (HER1, HER3, and HER4). These proteins are membrane bound and form homo- and hetero-dimers with HER2 and participate in regulating downstream signaling [10]. Recent literature has supplied direct evidence that HER4 plays a key role in modulating response to trastuzumab therapy [11]. Early in vitro studies using HER2 positive cell lines showed that transfection and over-expression of HER4 resulted in increased apoptosis [12, 13]. These studies provided the first mechanistic evidence that HER4 over-expression serves as a block to HER2 signaling activity, when HER2 and HER4 are co-over-expressed. Unlike HER2, HER4 over-expression appears to have an anti-proliferative and pro-apoptotic activity [14, 15]. In studies performed on human breast carcinoma, the reported prevalence of HER4 over-expression ranges from 12% to 82% in tumors and has been linked to both improved and poor clinical outcome, depending on antibody and study design [16-18]. This wide range of reported over-expression highlights a fundamental challenge of interpreting previous HER4 studies in breast carcinoma, which is the lack of a clinically validated standard anti-HER4 antibody and IHC scoring algorithm[11, 18, 19].

One potential reason for a lack of standardization in clinical IHC studies is the complex nature of HER4, which has four distinct isoforms secondary to proteolytic cleavage that can induce localization to multiple sub-cellular locations [20, 21]. Of the four isoforms of HER4, only one isoform is expressed in breast carcinoma (JM-a) [22, 23]. The expressed isoform can be membrane bound, or once proteolytically cleaved, can produce a soluble extra-cellular domain and a free intra-cellular domain. The cleavage site contributes to the unique localization and function of HER4 and likely plays a critical role in regulating HER2 positive carcinomas and the therapeutic response to HER2 over-expressing tumors[11, 18, 19, 24-28].

Recently a large number of HER4 antibodies were screened using both cell lines transfected with HER1, HER2, HER3, and HER4; and breast carcinoma samples [29]. The anti-HER4 clone E200 showed the greatest sensitivity and specificity for HER4 detection. In addition, this antibody showed a range of staining intensities in breast carcinoma cases, that was quantifiable and likely attributable to differences in HER4 expression status between patients. Based on these findings, the HER4 E200 clone was selected for use in the present study.

In this study, we set out to evaluate the predictive nature of HER4 over-expression in patients treated with trastuzumab therapy. To accomplish this we generated and standardized a novel IHC scoring algorithm for HER4 (H-Score). Utilization of this HER4 H-Score in conjunction with HER2 expression data, showed that patients that co-over-expressed both HER4 and HER2 showed a delay in development of metastasis (neoadjuvant population) and improved progression free survival (metastatic population). These findings demonstrate the clinical value of addition of HER4 expression data in the context of other standard markers including HER2, estrogen receptor (ER), progesterone receptor (PR) and Ki-67.

## RESULTS

### Determination of HER2 and HER4 Status in Neoadjuvant and Metastatic Trastuzumab Treated Cohorts

Distributions of clinical and pathologic characteristics of both the neoadjuvant and metastatic cohorts are presented in Table1. HER2 status was determined for each patient sample using multiple independent methodologies which included immunohistochemistry (IHC), in situ hybridization (ISH) [(Fluorescent (FISH) and Dual DNA (DISH)], and real time-quantitative PCR (RT-qPCR).

**Table 1:** Distribution of Clinical and Pathologic Characteristics of Study Population

	Neoadjuvant Cohort		Metastatic Cohort	
	no. of patients	%	no. of patients	%
AGE at diagnosis, years				
<40	7	14.9	4	12.12
40-55	16	34.0	20	60.61
>55	24	51.1	9	27.27
Tumor size, cm				
<2	8	17.0	6	18.18
2-5	31	66.0	17	51.52
>5	8	17.0	10	30.30
Tumor grade				
1&2	34	72.3	12	36.36
3	13	27.7	21	63.64
Lymph nodes				
Negative	34	72.3	7	21.21
Positive	13	27.7	26	78.79
HER2 over-expression				
Neg.	11	23.4	6	18.18
Pos.	36	76.6	27	81.82
HER4 over-expression				
Neg.	29	61.7	20	60.61
Pos.	18	38.3	13	39.39

In the neoadjuvant cohort, consensus in at least two out of the three methodologies (IHC, ISH, RT-qPCR) was required for classification of HER2 as over-expressed. In the neoadjuvant cohort, 36 of 47 patients demonstrated HER2 over-expression (Table1). Minor discrepancies were observed in 4 cases by IHC, which showed 2+ equivocal staining, however, all 4 cases were identified as amplified on *HER2* reflex ISH testing (Supplemental Table 1). Discrepancy by RT-qPCR were seen in 3 cases which were classified as *HER2* non-amplified, however these cases were 3+ by IHC and amplified by ISH (RT-qPCR scores 6.33, 6.75, and 6.9; Supplemental Table 1).

In the metastatic cohort, consensus in at least two out of the three methodologies resulted in classification of HER2 as over-expressed in 27 of 33 patients (Table1). No discrepancies were identified by any methodology in the HER2 over-expressed population. However, one case classified as non-over-expressed showed an equivocal 2+ IHC staining, this case was negative by both FISH and HER2 RT-qPCR score (1.7 FISH ratio and 6.59 HER2 RT-qPCR score).

**Figure 1:**
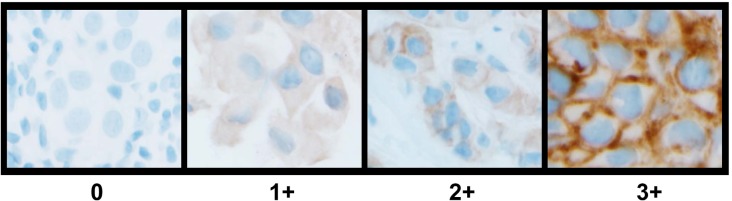
Representative images of HER4 (E200) IHC staining patterns and scoring in invasive breast carcinoma

**Figure 2:**
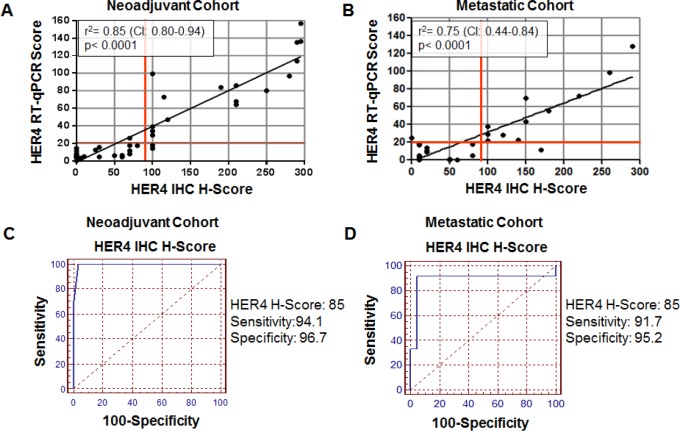
Correlation of HER4 (E200) IHC H-Score to *HER4* RT-qPCR A) Neoadjuvant cohort; B) Metastatic (adjuvant) cohort; C) ROC curve based determination of HER4 H-Score cut-off in the neoadjuvant cohort; D) ROC curve based determination of HER4 H-Score cut-off in the metastatic cohort.

Classification of HER4 expression status was determined by application of a pathologist based semi-quantitative IHC derived H-Score. Representative images of HER4 (E200) IHC staining patterns including membrane and cytoplasmic staining are shown in Figure 1. The analytical sensitivity of the IHC score was correlated with *HER4* RT-qPCR analysis of matched specimens. The correlation between *HER4* H-Score and *HER4* RT-qPCR was highly significant, with a correlation coefficient of r2= 0.85 (p<0.0001; CI: 0.80-0.94) for the neoadjuvant cohort (Figure 2A) and r2= 0.75 (p<0.0001; CI: 0.44-0.84) for the metastatic cohort (Figure 2B). Based on the significant correlation between RT-qPCR and *HER4* H-Score, ROC curve analysis was used to identify an H-Score value that corresponded to HER4 over-expression. Based on ROC curve analysis, an H-Score cut off of ≥85 for HER4 over-expression showed maximal sensitivity 94.1% (CI: 71.2- 99.0) and specificity 96.7% (CI: 88.3-100) in the neoadjuvant cohort (Figure 2C). Likewise, in the metastatic cohort, an H-Score cut off of ≥85 for HER4 over-expression showed maximal sensitivity 91.7% (CI: 68.8- 98.9) and specificity 95.2% (CI: 72.6- 99.1) (Figure 2D).

### Evaluation of Clinical Outcomes Using Solitary IHC HER2 or HER4 Expression Status

**Figure 3:**
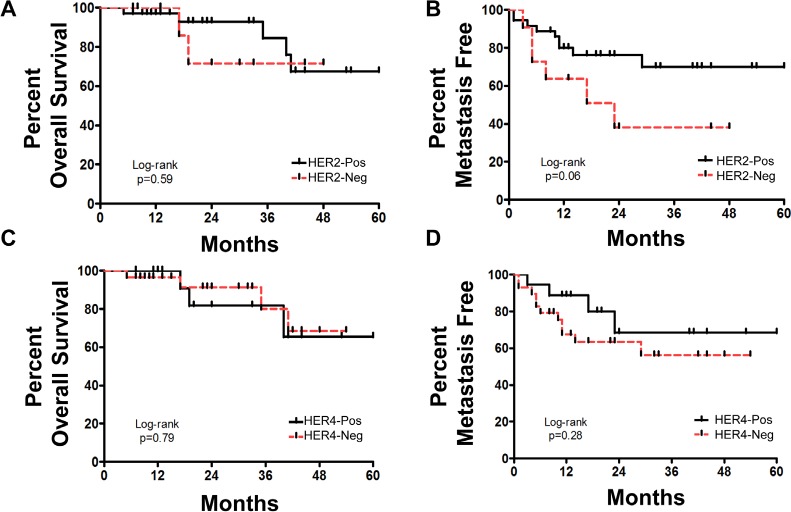
Neoadjuvant cohort response to trastuzumab as determined by HER2 or HER4 expression status A) Overall survival based on HER2 expression status; B) Percent metastasis free survival based on HER2 expression status; C) Overall survival based on HER4 expression status; D) Percent metastasis free survival based on HER4 expression status.

In the neoadjuvant cohort, three clinical outcome measures were evaluated which included complete pathologic response (CpR), overall survival (OS), and development of metastasis post trastuzumab therapy (Mets). Analysis based solely on HER2 expression status, showed no significant difference in CpR, OS, or development of Mets (p values: 0.30, 0.59, and 0.06 respectively) (Table 2). Similarly, analysis based solely on HER4 expression status, showed no significant difference in CpR, OS, or development of Mets (p-values: 0.56, 0.79, and 0.28 respectively) (Table 2). Kaplan-Meier plots for OS and development of METS based on HER2 expression status or HER4 expression status showed no significant predictive ability based on either single marker, plots are shown in Figure 3.

**Table 2:** Outcome measures including complete pathologic response, overall survival, and development of metastasis in the neo-adjuvant cohort segregated solely by HER2 or HER4 expression

		CpR	Non-CpR	Fisher's Exact	Survival	Non-Survival	Log-Rank	MET	No-MET	Log-Rank
HER2	Pos.	18	18	p=0.30	31	5	p=0.59	8	28	p=0.06
	Neg.	3	8		10	1		5	6	
HER4	Pos.	7	11	p=0.56	16	2	p=0.79	3	15	p=0.28
	Neg.	14	15		25	4		10	19	

Due to the low number of HER2 negative cases (6 out of 33) in the metastatic cohort, a statistical evaluation of clinical outcomes based solely on HER2 status was not sufficiently powered.

### Evaluation of Clinical Outcome using Combined HER2 and HER4 IHC Status

**Figure 4:**
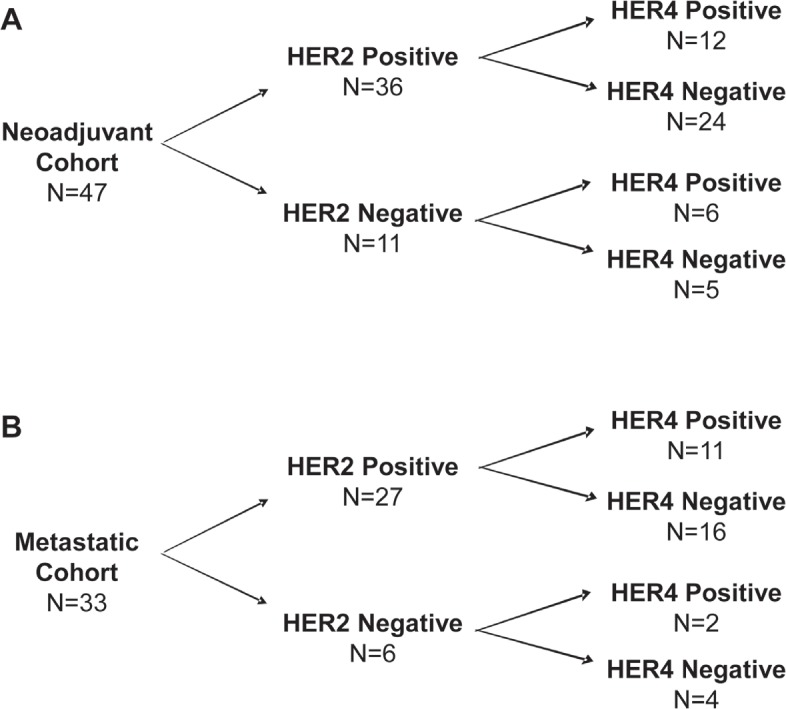
Summary of HER2 and HER4 expression in both study cohorts A) Neoadjuvant cohort; B) Metastatic cohort.

**Figure 5:**
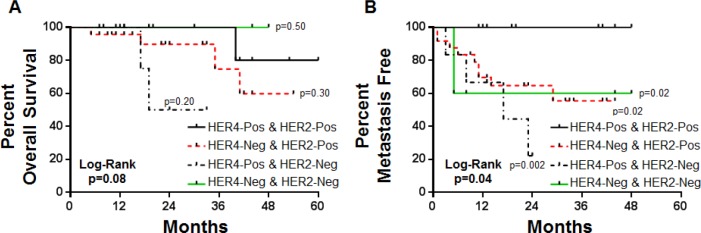
Kaplan-Meier plots for overall survival and development of metastasis in neoadjuvant trastuzumab treated cohort segregated by HER2 and HER4 co-expression patterns

In the neoadjuvant cohort, by IHC, 12 cases were positive for both HER2 and HER4, 24 cases were positive for HER2 and negative for HER4, 6 cases were negative for HER2 and positive for HER4, and 5 cases were negative for both HER2 and HER4 (Figure 4A). For CpR and OS, no statistical difference was observed for any HER2/HER4 combination (Table 3). However, for development of Mets, there was a significant difference between patients that co-over-expressed both HER2 and HER4 as compared to all other combinations of HER2/HER4 expression (p=0.002-0.02) (Table 3). Kaplan-Meier plots for OS and development of Mets based on HER2 and HER4 co-expression status showed co-over-expression of HER2 and HER4 resulted in a significant ability to predictive metastasis free survival but not OS, plots are shown in are shown in Figure 5A-B.

**Table 3:** Outcome measures including complete pathologic response, overall survival, and development of metastasis in the neo-adjuvant cohort segregated by HER2 and HER4 co-expression patterns

HER2 & HER4 Status	Number of Cases	CpR	Non-CpR	Fisher's Exact	Survival	Non-Survival	Log-Rank	MET	No-MET	Log-Rank
HER2 Pos. HER4 Pos.	12	5	7	n/a	11	1	n/a	0	12	n/a
HER2 Pos. HER4 Neg.	24	13	11	p=0.72	20	4	p=0.30	8	16	p=0.002
HER2 Neg. HER4 Pos.	6	2	4	p=>0.99	5	1	p=0.20	3	3	p=0.02
HER2 Neg. HER4 Neg.	5	1	4	p=0.60	5	0	p=0.50	2	3	p=0.02

**Figure 6:**
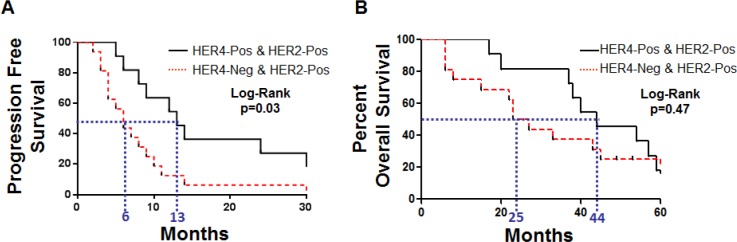
Kaplan-Meier plots for metatastic cohort A) Progression free survival and B) Overall survival.

In the metastatic cohort, by IHC, 11 cases were positive for both HER2 and HER4, 16 cases were positive for HER2 and negative for HER4, 2 cases were negative for HER2 and positive for HER4, and 4 cases were negative for both HER2 and HER4 (Figure 4B). Due to the low number of cases lacking HER2 over-expression, an evaluation of clinical outcomes in this population was not considered meaningful. However, clinical outcomes including progression free survival (PFS) and overall survival (OS) were evaluated for cases with HER2 over-expression in the metastatic cohort. For PFS, there was a significant difference between patients that co-over-expressed both HER2 and HER4 vs. patients that lacked HER4 over-expression (HER2 positive/HER4 negative) (p=0.03) (Figure 6A). Additionally, in this cohort, the median PFS improved from 6 months in cases lacking HER4 over-expression (HER2-Pos/HER4-Neg) to 13 months in cases with HER4 over-expression (HER2-Pos/HER4-Pos) (Figure 6A). Evaluation of OS in this cohort revealed a clear separation in the survival curves between patients with co-over-expression of HER2 and HER4 vs. patients that lacked HER4 over-expression (HER2-Pos and HER4-Neg); this difference did not reach statistical significance (p=0.47) (Figure 6B). However, in this metastatic cohort, the median OS improved from 25 months in cases that lacked HER4 over-expression to 44 months in cases with co-over-expression of HER4 and HER2 (Figure 6B).

**Table 4:** Tumor response, Time to progression and Overall survival in Metastatic Trastuzumab treated cohort segregated by HER2 and HER4 co-expression patterns

HER2 & HER4 Status	Number of Cases	PFS	Non-PFS	Log-Rank	Survival	Non-Survival	Log-Rank
HER2 Pos. HER4 Pos.	11	2	9	n/a	9	2	n/a
HER2 Pos. HER4 Neg.	16	10	6	p=0.03	6	10	p=0.47

### Evaluation of Clinical Outcome using Combined HER2, HER4 and ER IHC based Expression Status

In our analysis, we observed an increased association between HER4 expression and ER-positive status in both the neoadjuvant and metastatic cohort. In the neoadjuvant cohort, ER expression was observed in 11 out of 12 cases that over-expressed HER4 (HER2-Pos and HER4-Pos) and 13 out of 24 cases that lacked HER4 over-expression (HER2-Pos and HER4-Neg). Similarly, in the metastatic cohort, ER expression was observed in 10 out of 11 cases that over-expressed HER4 (HER2-Pos and HER4-Pos) and 8 out of 16 cases that lacked HER4 over-expression (HER2-Pos and HER4-Neg) (Supplemental Table 2). To determine if ER expression could influence the predictive nature of HER4, we directly compared HER2-Pos/ER-Pos/HER4-Pos and HER2-Pos/ER-Pos/HER4-Neg outcomes in both the neoadjuvant and metastatic cohorts. Results show that in a uniform ER expressing population, over-expression of HER4 retained a significant ability to predict Met free survival in the neoadjuvant cohort and predict both PFS and OS in the metastatic cohort (Supplemental Figure 1A-C).

## DISCUSSION

While HER2 is a well characterized predictive marker in breast carcinoma, the predictive marker status of HER4 is largely unconfirmed. Previous HER4 IHC studies in breast cancer have implicated this target as a potential new predictive biomarker in patients receiving anti-HER2 therapy. However, findings from these previous studies are diminished due to the lack of consistent antibody and lack of controls showing antibody specificity for HER4. In addition, alternative splicing of HER4-encoded products results in multiple isoforms that can be present in multiple cellular locations. Due to these challenges, the role of HER4 as a predictive marker in trastuzumab treated breast cancer remains unconfirmed.

In this study, we evaluated whether HER4 can serve as a potential predictive biomarker in trastuzumab treated breast carcinoma. We accomplished this by first, utilizing a single HER4 antibody (E200) which was recently stringently tested and validated in breast carcinoma and second, by developing a standardized semi-quantitative H-Score system for determining HER4 expression status in breast carcinoma. Based on the recently published work by Jay et al., we chose to utilize the HER4 E200 clone to develop our clinical based IHC H-Score expression assay. We evaluated our HER4 H-Score in two invasive breast carcinoma cohorts that received trastuzumab 1) in a neoadjuvant setting (n=47) and 2) for metastatic disease (n=33). These two cohorts showed significant overlap in clinical and pathological characteristics. The only exceptions included, the neoadjuvant cohort was older at presentation and presented with a lower tumor grade. However, the HER2 and HER4 expression profiles and percent showing over-expression were highly consistent between the two cohorts.

To circumvent problems observed in previous qualitative HER4 studies, we chose to standardize a single antibody and use a semi-quantitative scoring system for HER4 expression in breast carcinoma. To assess the analytical sensitivity of the HER4 IHC H-Score, we utilized RT-qPCR for HER4 as a standard for comparison, due to the high sensitivity of the qPCR assay and our previous success with validation of a HER2 RT-qPCR assay [30]. Like our previous HER2 based RT-qPCR, HER4 amplification was successfully quantified using this assay. The HER4 RT-qPCR values were directly correlated to HER4 H-Scores and a significant correlation was identified. Furthermore, ROC curve analysis utilizing HER4 RT-qPCR and HER4 H-Scores demonstrated an optimal cut off for HER4 over-expression status (H-Score of greater than or equal to 85). This value was consistent between both the neoadjuvant and metastatic cohorts. In situ hybridization was not performed for HER4 due to the lack of repeat-deleted probes available for this target. Future studies utilizing ISH are warranted and a novel HER4 ISH probe is in development for this purpose.

Both cohorts were evaluated for clinical and pathologic outcome measures based on combinations of HER2 and HER4 expression status. Analysis based on HER2 or HER4 expression status alone in the neoadjuvant cohort failed to predict CpR, OS, or Met free survival. This single marker analysis was not relevant in the metastatic cohort due to lack of a significant number of HER2 negative cases for a meaningful comparison.

While HER2 and HER4 single gene over-expression failed to serve as a predictive marker for trastuzumab therapy, paired analysis improved the predictive nature of these markers. As demonstrated in the neoadjvant cohort, co-over-expression of HER2 and HER4 showed a significantly longer period of metastasis free survival. Likewise, in the metastatic cohort, patients with co-over-expression of HER2 and HER4 demonstrated a longer median PFS (7 month improvement) and an improved median OS (19 month improvement).

While our results implicate HER2 and HER4 dual over-expression is directly linked to predicting post trastuzumab outcome, it is possible that other markers including ER could play a critical role in influencing this association. To address this issue, we analyzed the ER expression status in our two cohorts and compared ER expression in the context of the presence or absence of HER4 over-expression. We observed that there was an increased association between HER4 expression and co-ER expression in both cohorts (Supplemental Table 2). However, further analysis revealed that ER expression status failed to alter the significant difference in outcomes seen based on the presence or absence of HER4 over-expression (Supplemental Figure 1). This finding does not exclude the possibility that an additional biomarker modulates the predictive ability of HER4, but it does provide direct evidence that the predictive ability of HER4 is not a result of co-expression of ER.

The current study is limited by the retrospective study design, relatively small patient populations and enrichment by HER2 positive cases. In spite of these limitations, we were able to generate and standardize a novel H-Score system for determining HER4 expression status and were able to utilize this new scoring system, in conjunction with HER2 expression status, to significantly segregate patients based on quantifiable outcome measures. Expanding these studies to larger cohorts may reveal further significant predictive ability for metrics such as overall survival, which in the neoadjuvant cohort trended toward but did not reach statistical significance (p=0.08). Finally, while direct comparison between equal populations of HER2 positive and negative patients would be ideal, this is not possible due to the toxicity of trastuzumab and the current reservation of this drug for HER2 positive cases, thereby excluding the recruitment of an equal number of HER2-negative patients. While limitations were present in this study, one of the great strengths of this study includes use of a neoadjuvant cohort, which allowed definitive outcome measures post trastuzumab treatment via direct visualization/identification of tumor size at time of resection.

While HER2 negative patients are not candidates for trastuzumab therapy, it is interesting that of the 47 patients that received trastuzumab in the neo-adjuvant cohort, 11 cases (23%) did not show IHC criteria for HER2 over-expression on pretreatment biopsy material based on ASCO/CAP guidelines. Of the 11 cases, 6 were equivocal by IHC and 9 cases were equivocal by either FISH or DISH (Supplemental Table 3). The remaining 2 cases which were negative by FISH and DISH, were patients that transferred care and had a positive HER2 report per outside laboratory testing but were negative by in-house testing. A second interesting finding was that the HER2 RT-qPCR assay identified all 11 of these cases as negative (RT-qPCR score <7.0) (Supplemental Table 3).

## CONCLUSION

In conclusion, we addressed a major current clinical need, which is identification of a potential novel predictive biomarker in breast carcinoma. This was accomplished by selecting the partially characterized marker HER4 and developing a single antibody diagnostic assay utilizing a standardized pathologist based IHC scoring system. This scoring system was validated with a highly sensitive RT-qPCR assay and was applied to two separate breast cancer cohorts. In both the neoadjuvant and metastatic trastuzumab treated cohorts, HER4 over-expression, in conjunction with over-expression of HER2, showed significant predictive ability in selecting patients with improved metastasis free survival, progression free survival, and overall survival. Use of a dual HER2/HER4 IHC assay at time of diagnosis could improve outcome prediction in patients treated with trastuzumab and ultimately result in improved physician-patient counseling on disease course.

## MATERIAL AND METHODS

### Study Population

Following approval of a registry (09-226) by the Cleveland Clinic Institutional Review Board, the electronic medical records for all patients that had received trastuzumab at the Cleveland Clinic from 1/1998 to 12/2010 were reviewed for potential inclusion (445 patients). Of the 445 cases, 47 satisfied inclusion criteria for analysis of subjects in the neoadjuvant setting, which included a diagnosis of primary invasive breast carcinoma, neoadjuvant trastuzumab therapy, and a pre-treatment biopsy performed at the Cleveland Clinic. Of the 445 cases, 33 patients satisfied inclusion criteria for metastatic disease setting, which included a diagnosis of primary invasive breast carcinoma, trastuzumab therapy for metastatic disease without prior trastuzumab exposure and resection of the primary tumor performed at the Cleveland Clinic.

### Immunohistochemistry for HER2 and HER4

IHC analysis was performed as previously described [30]. Briefly, 4 μm formalin fixed paraffin embedded (FFPE) tissue sections were used for automated staining carried out on the Ventana Benchmark XT system (Ventana Medical Systems, Tucson, AZ) using the PATHWAY® anti-HER2/neu (4B5) rabbit monoclonal primary antibody (Ventana Medical Systems, Tucson, AZ), Anti-HER4 (E200) rabbit monoclonal primary antibody (Epitomics, Burlingame, CA) and ultraVIEW DAB detection kit (Ventana Medical Systems, Tucson, AZ). Standard blocks containing four human breast cancer cell lines with known intensity scores of 0, 1+, 2+, and 3+ were used as appropriate controls for HER2 IHC. Negative controls were performed by omitting the primary antibody.

### Immunohistochemical analysis for HER2, HER4, ER, and PR

All H&E and immune-labeled slides were reviewed by two pathologists (BP and EM) who were blinded to HER2/HER4 scores obtained by other methods or the patient outcomes. A subset of sections was reviewed by an additional pathologist (RRT).

HER2 IHC was scored according to ASCO/CAP HER2 testing guidelines [31]. Briefly, a 3+ staining pattern is interpreted as positive for HER2 and is defined as uniform intense membrane staining of 30% of invasive tumor cells. A 2+ staining pattern is interpreted as equivocal for HER2 and is defined as complete membrane staining that is either nonuniform or weak in intensity but with obvious circumferential distribution in at least 10% of cells. A 0 or 1+ staining pattern is interpreted as negative for HER2 (ERBB2) and is defined as no staining (0) or weak, incomplete membrane staining (1+) in any proportion of the tumor cells.

Consensus review of stain pattern and intensity for HER4 (E200) IHC resulted in standardization and generation of a traditional pathologist based H-score. Briefly, in the invasive carcinoma regions of the histologic sections, the chromogenic immunolabeling was systematically categorized into four groups: 0 (no membrane or cytoplasmic labeling), 1+ (weak cytoplasmic labeling), 2+ (weak membrane and/or strong cytoplasmic labeling), and 3+ (strong membrane (observable with 10x objective); with or without cytoplasmic staining). A single manual H-score based on a scale of 0 to 300 was generated for each labeled section by taking the sum of the percentage of cells labeling 1+, double the percentage of cells labeling 2+, and triple the percentage of cells labeling 3+ (H-Score= ((%3+) × 3) + ((%2+) × 2) + (%1+)).

IHC results for estrogen receptor (ER) and progesterone receptor (PR) were obtained from clinically reported IHC results located in electronic medical record.

Images in this report were obtained from an Olympus BX40 microscope with an Olympus DP72 microscope digital camera (Olympus, Tokyo, Japan). Image capture settings and acquisition was performed using MetaMorph® software (Olympus, Tokyo, Japan).

### Real Time-Quantitative PCR (RT-qPCR)

HER2 and ERBB4 (HER4) RT-qPCR assays were performed as previously published [30]. Briefly, all unstained slides were cut fresh (one 4μm slide for H&E and five 10μm slides for RT-qPCR). From the 10μm unstained slides, 4 were macro-dissected, guided by location of tumor identified by H&E. RNA extraction was performed following macro-dissection using High Pure RNA Paraffin Kit (Roche Applied Science, Indianapolis, IN). RT-qPCR was carried out in triplicate using a TaqMan® RNA-to-CT™ 1-Step master mixtures Kit with primers and monocolor hydrolysis probes Hs01001580_m1 (HER2), Hs00955525_m1 (HER4), Hs00955525_m1 (B2M), Hs00984230_m1 (GAPDH), and Hs03929097_g1 (TFRC) (Applied Biosystems, Foster City, CA). RT-qPCR was performed using a LightCycler 480 II machine (Roche Applied Science, Penzberg, Germany) running LightCycler® 480 SW 1.5 software according to the manufacturer's instructions. The RT-qPCR cycling conditions for all the genes were as follows: 48°C for 15 min, 95°C for 10 min, followed by 50 cycles of 95°C for 15 s, 60°C for 1 min, followed by 37°C for 1 min. PCR products were subjected to electrophoresis on agarose gel to confirm the absence of nonspecific PCR products. Analysis of the crossing threshold point (CT) for the amplification curves for each specimen was determined by the second derivative maximum method [32]. Absolute quantitation was performed with an in-run standard curve. Results were expressed as the ratio of HER2 to reference gene copies. Combination of two control genes, B2M and GAPDH, provided superior separation of control populations. Therefore B2M plus GAPDH were utilized as reference genes for this study. All results were normalized against calibrator RNA extracted from the MCF7 breast cancer cell line.

### HER2 FISH and DISH

HER2 FISH or dual DNA in situ hybridization (DISH) analysis was performed on each case of breast carcinoma at the time of diagnosis utilizing paraffin-embedded tissues with the PathVysion HER2 DNA Probe Kit (Abbott Molecular-Vysis, Downers Grove, IL); some cases were also evaluated using the INFORM HER2 Dual ISH kit (Ventana Medical Systems, Tucson, AZ) according to the manufacturer's instructions as specified in the package insert. Each FDA kit contained specific DNA probes to the HER2 gene locus and to the alpha centromeric region of chromosome 17. The probe signals were counted in 40 tumor nuclei per case under a fluorescence microscope with appropriate filters. Results were reported as the average ratio of HER2 signals to chromosome 17 signals in non-overlapping interphase invasive carcinoma nuclei.

### Statistics

H-Scores and RT-qPCR scores were subjected to analysis using Prism Version 5.02 (GraphPad V6.0, La Jolla, CA, USA) by selecting correlation analysis with Pearson test and the two-tailed p-value option. ROC curve analysis was performed using MedCalc® V8.0.1.0 (MedCalc Software, Mariakerke, Belgium). Kaplan-Meier data and statistics were subjected to analysis using Prism Version 5.02 (GraphPad V6.0, La Jolla, CA, USA) by selecting log-rank test.

## Supplementary Table and Figures




