# Supplementary material for: HER4 expression status correlates with improved outcome in both neoadjuvant and adjuvant Trastuzumab treated invasive breast carcinoma
Source: Oncotarget. 2013 Aug 26;4(10):1662–72. doi: 10.18632/oncotarget.1232 (PMC3858553; doi:10.18632/oncotarget.1232)
Supplement: Supplementary file 1 [file oncotarget-04-1662-s001.pdf]

HER4 Expression Status Correlates with Improved Outcome in Both Neoadjuvant and Adjuvant Trastuzumab Treated Invasive Breast Carcinoma – Portier et al

Supplemental Table 1: Neoaduvant trastuzumab treated cohort, cases with discrepancy in HER2 status by one of three detection methods.

Neoadjuvant cohort: HER2 Positive Cases

HER2 IHC	*HER2* FISH Ratio	*HER2* DISH Ratio	*HER2* RT-qPCR
2	3.0	3.0	39.03
2	3.0	2.3	72.72
2	2.3	2.3	136.18
2	2.9	2.2	**6.33
3	14.6	20.2	39.35
3	7.5	9.3	113.17
3	6.2	8.0	44.52
3	8.3	9.5	51.85
3	5.6	7.0	19.07
3	10.1	6.4	30.71
3	3.7	11.0	38.13
3	6.6	6.8	42.61
3	2.7	2.3	24.67
3	4.1	8.0	7.78
3	5.8	14.3	78.56
3	9.6	11.2	38.58
3	11.1	9.7	65.12
3	6.6	11.0	54.55
3	6.7	7.5	109.47
3	7.5	6.5	57.16
3	5.5	6.7	34.03
3	16.7	16.6	123.78
3	7.2	6.9	46.21
3	8.4	8.2	66.2
3	9.9	10.3	34.69
3	9.5	8.9	81.68
3	13.5	9.5	31.47
3	6	5.2	126.94
3	9.1	9.4	18.14
3	5.9	7.5	75.76
3	5.6	6.0	72.65
3	3.7	4.5	**6.75
3	8.4	7.2	25.31
3	5.1	6.6	**6.9
3	10.2	9.2	70.6
3	8.7	7.9	81.49

Grey box: Equivocal IHC cases reflex tested by ISH and RT-qPCR

**: Discrepancy between RT-qPCR and IHC/ISH HER2 detection methodologies

Supplemental Table 2: ER and PR expression status in cases both with and without HER4 over-expression in the neoadjuvant and metastatic cohorts

Neoadjuvant Cohort: HER2 Positive and HER4 Positive

ER	PR	IHC HER2 (FISH Ratio)	HER4 IHC H-Score
Positive	Positive	3	210
Positive	Positive	3	100
Positive	Positive	2 (3.0)	100
Positive	Positive	3	290
Positive	Positive	3	210
Positive	Positive	3	210
Positive	Positive	3	190
Positive	Positive	2 (3.0)	115
Positive	Positive	2 (2.3)	295
Positive	Negative	3	120
Negative	Negative	3	100
Positive	Negative	2 (2.9)	290

Neoadjuvant Cohort: HER2 Positive and HER4 Negative

ER	PR	IHC HER2 (FISH Ratio)	HER4 IHC H-Score
Positive	Positive	3	0
Negative	Negative	3	10
Positive	Negative	3	0
Negative	Negative	3	30
Negative	Negative	3	60
Negative	Negative	3	0
Positive	Positive	3	70
Negative	Negative	3	0
Positive	Positive	3	0
Negative	Negative	3	0
Positive	Positive	3	70
Negative	Negative	3	0
Negative	Negative	3	0
Negative	Negative	3	5
Negative	Negative	3	5
Positive	Positive	3	0
Positive	Negative	3	25
Positive	Negative	3	30
Positive	Positive	3	0
Negative	Negative	3	80

Positive	Positive	3	70
Positive	Positive	3	70
Positive	Positive	3	60
Positive	Positive	3	0

Metastatic Cohort: HER2 Positive and HER4 Positive

ER	PR	IHC HER2 (FISH Ratio)	HER4 IHC H-Score
Positive	Negative	3	100
Positive	Positive	3	180
Positive	Positive	3	290
Positive	Positive	3	150
Positive	Positive	3	100
Positive	Positive	3	260
Positive	Positive	3	220
Positive	Positive	3	120
Negative	Negative	3	100
Positive	Positive	3	170
Positive	Positive	3	140

Metastatic Cohort: HER2 Positive and HER4 Negative

ER	PR	IHC HER2 (FISH Ratio)	HER4 IHC H-Score
Positive	Positive	3	80
Negative	Negative	3	80
Negative	Negative	3	10
Positive	Negative	3	10
Negative	Negative	3	50
Negative	Negative	3	10
Positive	Positive	3	10
Positive	Negative	3	10
Negative	Negative	3	20
Positive	Positive	3	20
Positive	Positive	3	20
Positive	Negative	3	10
Negative	Negative	3	10
Negative	Negative	3	10
Negative	Negative	3	0
Positive	Positive	3	10

Supplemental Table 3: IHC, FISH, DISH, and RT-PCR results for HER2 in the eleven neoadjuvant cohort cases classified as HER2-Negative in this study

HER2 Negative Cases (Neoadjuvant Cohort)

Number of Cases	IHC HER2	HER2 FISH Ratio	HER2 DISH Ratio	HER2 RT-qPCR
1	2	1.0	1.0	5.73
2	2	1.8	1.2	4.02
3	2	1.8	1.7	3.63
4	2	1.9	1.0	6.7
5	2	1.6	2.0	1.22
6	2	1.8	1.4	6.04
7	1	1.9	2.0	4.57
8	1	2.0	2.2	2.85
9	1	2.1	2.0	1.97
10	1	2.0	1.8	2.44
11	0	1.3	1.2	5.55

*Grey boxes= Negative test result
