# Supplementary material for: HER4 expression status correlates with improved outcome in both neoadjuvant and adjuvant Trastuzumab treated invasive breast carcinoma
Source: Oncotarget. 2013 Aug 26;4(10):1662–72. doi: 10.18632/oncotarget.1232 (PMC3858553; doi:10.18632/oncotarget.1232)
Supplement: Supplementary file 2 [file oncotarget-04-1662-s002.pdf]

HER4 Expression Status Correlates with Improved Outcome in Both Neoadjuvant and Adjuvant Trastuzumab Treated Invasive Breast Carcinoma – Portier et al

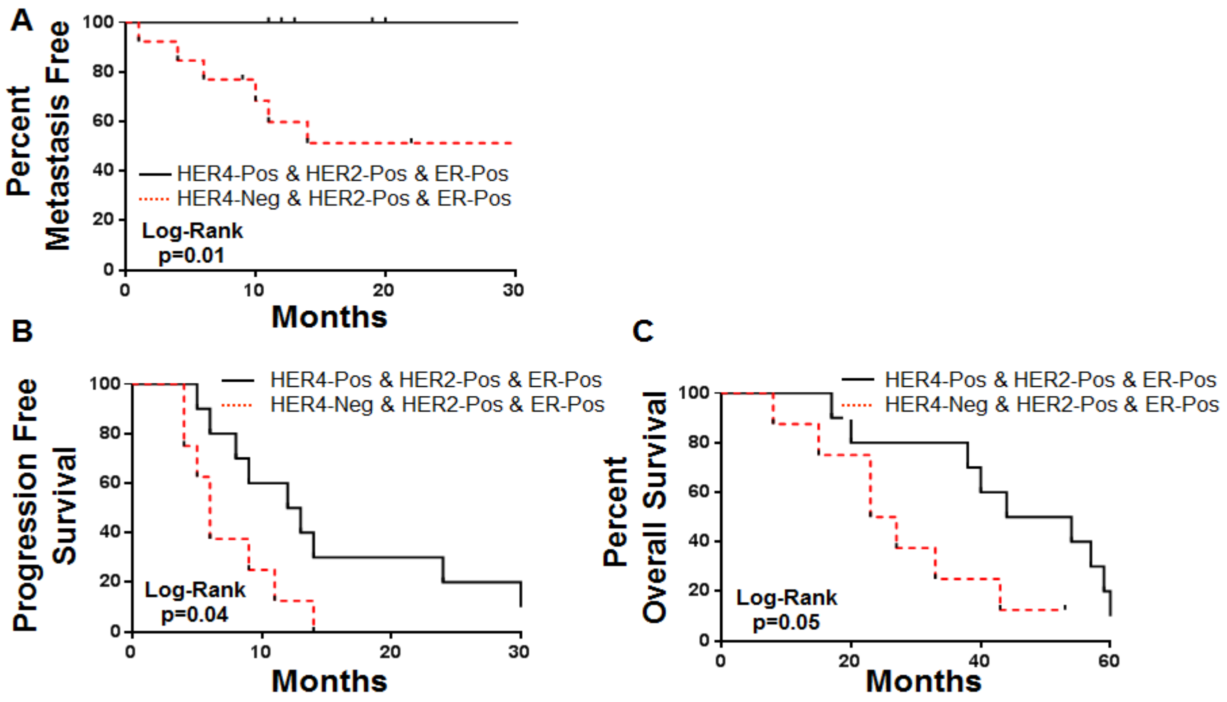

Supplemental Figure1 : Kaplan-Meier plots comparing co-expression of ER in both the neoadjuvant and metastatic cohorts A) metastasis free survival in neoadjuvant cohort; B) Progression free survival in metastatic cohort; C) Overall survival in metastatic cohort
